# Electrochemical Oxidation of Cysteine at a Film Gold Modified Carbon Fiber Microelectrode Its Application in a Flow—Through Voltammetric Sensor

**DOI:** 10.3390/s120303562

**Published:** 2012-03-14

**Authors:** Lai-Hao Wang, Wen-Shiuan Huang

**Affiliations:** Department of Medical Chemistry, Chia Nan University of Pharmacy and Science, 60 Erh-Jen Road, Section 1, Jen Te, Tainan 71743, Taiwan; E-Mail: michellehuang@ritdisplay.com

**Keywords:** micro Au-modified carbon fiber electrode, pulse amperometric detection, cysteine

## Abstract

A flow-electrolytical cell containing a strand of micro Au modified carbon fiber electrodes (CFE) has been designedand characterized for use in a voltammatric detector for detecting cysteine using high-performance liquid chromatography. Cysteine is more efficiently electrochemical oxidized on a Au /CFE than a bare gold and carbon fiber electrode. The possible reaction mechanism of the oxidation process is described from the relations to scan rate, peak potentials and currents. For the pulse mode, and measurements with suitable experimental parameters, a linear concentration from 0.5 to 5.0 mg·L^−1^ was found. The limit of quantification for cysteine was below 60 ng·mL^−1^.

## 1. Introduction

The sulfhydryl (-SH) group of cysteine plays a key role in the biological activity of proteins and enzymes. It is responsible for disulfide bridges in peptides and proteins. l-Cysteine (Cys, l-2-amino-3-mercaptopropionic acid) is a biologically important sulfur-containing amino acid which is involved in a variety of important cellular functions, including protein synthesis, detoxification and metabolism [1]. The biological reactions of cysteine are accompanied by SH-SS exchange reactions and the conversion of the disulphide into a dithiol group [2]. Thioproline (thiazolidine 4-carboxylic acid) is metabolized *in vitro* by liver mitochondria to produce the ring-opened N-formylcysteine; a reaction reported to be catalysed by a specific dehydrogenase described the *in vivo* conversion of thioproline to cysteine, the reaction presumably occurring via N-formylcysteine [3].

Since cysteine itself lacks a strong chromophore, determining its presence/concentration by absorbance measurements is very difficult. Spectrophotometric detection is based on derivatization with cromogenic reagents in order to allow its detection by absorption spectrometry [4]. Many electrochemical strategies have been reported including chemically modified graphite electrodes [2,5–7] such as with cobalt (II) cyclohexylbutyate, praseodymium hexacyanoferrate, and Co(II)-Y zeolite modified graphite electrode; and using Nile blue A as a mediator at a glassy carbon electrode for determination of l-cysteine; Hg thin film sensor [8], biosensors based on electrodes modified with enzymes such as tyrosinase, laccase, l-cysteine desulfhydrase [9–11]. On the basis of the presence of the sulphuryl (-SH) function group in the structure of cysteine, its voltammetric adsorption and desorption has been investigated at a bare gold electrode [12,13] and composite film modified electrode with Au nanoparticles dispersed in Nafion [14]. Pulsed electrochemical detection (PED) is based on the application of repetitive multistep potential-time (*E*-*t*) waveforms to a noble metal electrode that manage the sequential processes of amperometric detection combined with pulsed potential cleaning. In order to improve the selectivity and sensitivity of determination of cysteine, alternative methods such as high-performance liquid chromatography or flow injection with pulsed electrochemical detection employing a gold working electrode have been published in the literature [15–18]. Due to the advantages of microelectrodes and ultramicroelectrodes their use in electrochemical studies has been an important area of recent years [19]. Carbon fibers belong to the electrodic materials most commonly used in the construction of microelectrodes. The main research topics were dealing with a mercury monolayer [20,21], hydro-coated glutamate [22] and gold [23] modified carbon fiber electrodes. These electrodes were constructed for capillary electrophoresis [24–28], liquid chromatography [29,30] to detect amino acids. The main advantages of these devices are smaller dead volume (dead space, void volume) of the device, a more convenient signal to noise ratio, and a reduced requirement of the supporting electrolyte in the solution. In this study we describe the construction of a disposable electrode sensor, composed of gold deposited on a carbon fiber substrate, for the high-performance liquid chromatography and the pulsed amperometric detection of cysteine.

## 2. Experimental Section

### 2.1. Apparatus and Materials

Voltammetric measurements were performed using an electrochemical trace analyzer (Model 394; EG&G Princeton Applied Research, Princeton, NJ, USA). A high-performance liquid chromatography (HPLC) system (LC-10 AD_vp_; Shimadzu, Kyoto, Japan) containing a Rheodyne 7125 injection valve with a 20-μL sample loop coupled to an amperometric detector (Decade II; Antec (Leyden) B.V., Zoeterwoude, The Netherlands). The flow cell was designed with the following electrodes: an Ag/AgCl/0.1 M KCl reference electrode (BAS), a stainless steel auxiliary electrode, and a gold modified carbon fiber electrode (length 8 cm, i.d. 7.54 μm) as working electrode for detecting cysteine. All solvents and analytes were filtered through 0.45-μm cellulose acetate and polyvinylidene fluoride syringe membrane filters, respectively. Chromatograms of cysteine were registered and peak height was calculated using a chromatogram data integrator (Scientific Information Service Corp., Davis, CA, USA). The samples of l-cysteine and hydrogen tetrachloroaurate(III) trihydrate (HAuCl_4_·3H_2_O) were purchased from Sigma (St. Louis, MO, USA) and Alfa Aesar (Ward Hill, MA, USA), respectively. A bundle of carbon fibers (polyacrylonitrile, PAN type) with 7.54 μm diameter obtained from the Formosa Synthetic Fiber Research Institute (Yunlin, Taiwan). All other reagents were locally purchased and of analytical grade.

### 2.2. Preparation of Thin-Film Gold Carbon Fiber Micro-Electrode for Voltammetric Measurements

A typical carbon fiber micro-electrode preparation procedure was as follows: a bundle of carbon fibers was connected together with a slender copper wire to ensure the electric contact the carbon fiber. The carbon fiber micro-electrode was placed in the tube containing HAuCl_4_ solution. The modified of Au/CFE was electrolytically plated with gold metal ion from 10 mL of 0.1 M acetate buffer (pH 4.97) that was 1.0 × 10^−3^ to 6 × 10^−3^ M HAuCl_4_ solution, respectively. Plating time was 4, 6, 8 and 9 min. respectively, by potential scan between −1.0 V and +1.0 V (*vs.* Ag/AgCl) (at 10 mV/s). The two voltammetric techniques, differential pulse voltammetry and cyclic voltammetry, were all performed on an Au/CFE electrode. Voltammograms of cysteine were taken on an Au/CFE electrode in a lithium perchlorate (pH 6.01), acetate buffer (pH 4.31), phosphate buffer solutions (pH 2.11 and 6.38) and Britton and Robinson buffer solutions (pH 1.82–8.05).

### 2.3. Construction of a Voltammetric Sensor for LC-PAD

The bare carbon fiber working electrode was fabricated by the following steps: (1) a single fiber was separated from a bundle of carbon fibers; (2) rational 8, 16, 32 individual fibers were rubbed together into a bundle by hand; (3) a welding torch was used to melt soldering tin (i.d. 1.0 mm; 60% Sn and 40% Pb; melt point 183–190 °C) into a globule; then one terminal of the bundle of fibers was combined with a copper wire (i.d. 0.15 mm) using the melting globule. The bare carbon fiber had gold deposited on its surface then it was inserted into one end of a Teflon tube and sealed with acrylic resin (obtained from Struers). Pulsed amperometric detection was achieved in a home-made flow through cell prepared in our laboratory as previously described [29] to detect cysteine. RP-HPLC was performed on a ThermoQuest Hypersil SCX column (particle size 5 μm, 250 mm × 4.6 mm i.d.) eluted with methanol-water (20:80, v/v, containing 10 mM acetate buffer, pH 4.65) as the mobile phase at flow rate of 0.5 mL/min.

## 3. Results and Discussion

### 3.1. Electrochemical Behavior of Cysteine at Au/CFE Electrode

Cysteine can be oxidized to the corresponding disulfide according to the following reaction:

$$2\;\text{RSH}⇄\text{RSSR}+2e^{-}+2H^{+}$$

**Figure 1.**
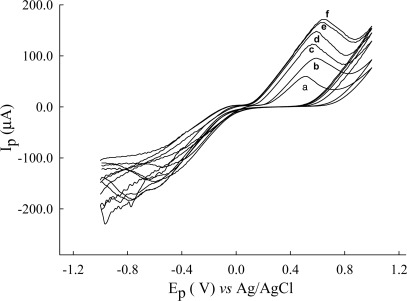
The growth patterns for a Au -coated carbon fibre (CFE), deposited from 4 mM HAuCl_4_ (Hydrogen tetrachloroaurate (III) trihydrate) in 0.1 M acetate buffer (pH 4.97) solution by continuous scan cyclic voltammetry (**a**) the first scan (**b**) the second scan (**c**) third scan (**d**) fourth scan (**e**) fifth scan (**f**) sixth scan, from −1.0 V to 1.0 V on a carbon fiber microelectrode (44.34 μm^2^ surface area), scan rate, 100 mV/s.

The cysteine-cystine system is not reversible at a platinum electrode, solely because of the slowness of the electrode reaction [31]. In order to achieve the optimum conditions for cysteine determination, there are several factors such as pH, supporting electrolytes, and working electrode which should be considered. The effect of pH of Britton-Robinson buffer as supporting electrolyte has been studied in the range from 1.82 to 8.05. Gold catalyst is usually obtained from solutions of HAuCl_4_ and its salts by chemical or electrochemical deposition. During deposition of a gold catalyst on a carrier it was found as the surface area and possibly the specific activity of gold depend on the substrate. In this study, two kinds of working electrodes that is microparticles of gold deposited on the carbon fiber electrode (Au/CFE) and a bare gold electrode (Au) were investigated. A typical example of the result of the cyclic voltammograms, the growth patterns for an Au-coated carbon fiber (CFE), obtained for the electrochemical growth of Au particles on a CFE can be seen in Figure 1.

**Figure 2.**
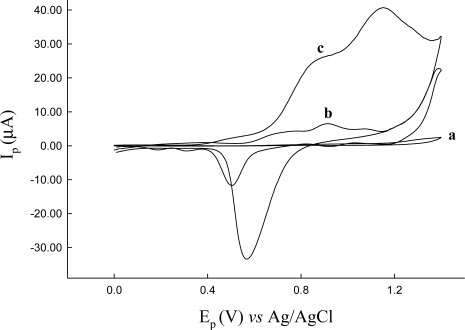
Cyclic voltammograms of cysteine (4 mg·L^−1^) in Britton-Robinson buffer pH 4.86: (**a**) at the bare CFE; (**b**) at the bare Au (i.d. 3 mm); (**c**) at Au modified CFE. Scan rate at 10 mV/s.

The peak current increased with scan numbers and current difference from first to fifth scan was larger than from sixth to tenth. The scans beyond the sixth scan have a small current difference. Figure 2 shows the electrochemical oxidation of cysteine (4 mg·L^−1^) at bare CFE, bare Au and the Au/CFE. It is shown that no obvious anodic peaks can be observed on CFE, and one peak 0.910 V, 6.51 μA is seen at a bare Au electrode. However, on the Au/CFE two well-defined oxidation peaks (peak 1 at 0.835 V, 24.4 μA and peak 2 at 1.15 V, 40.7 μA) were exhibited at pH 4.86 and a scan rate of 10 mV/s. The Au nanoparticles serve as large surface area platforms for sulfhydryl groups that interact with cysteine. Thus, the apparent found that peak current of Au/CFE was higher than with the CFE and bare Au electrode.

**Figure 3.**
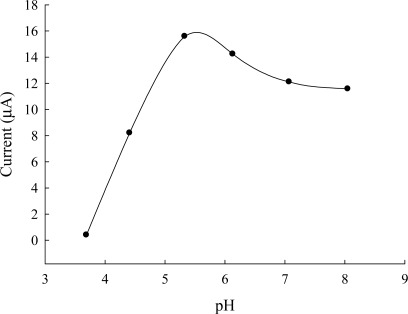
The effect of pH on the response current of cysteine (1.2 mg·L^−1^) in Britton-Robinson buffer at Au modified CFE; CV scan rate, 50 mV/s.

The relation between the peak current and pH for Britton-Robinson buffer is the plot of I_p_ *vs*. pH and depicted in Figure 3.

**Figure 4.**
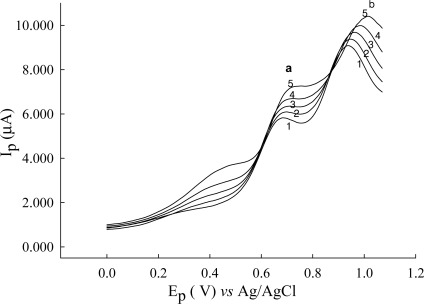
DPV obtained to construction calibration plot for cysteine at an Au/CFE. The peak potential and current values were: (1) with 4 mg·L^−1^ of cysteine at a (0.684 V, 5.80 μA), b (0.939 V, 9.04 μA); (2) with 8 mg·L^−1^of cysteine at a (0.693 V, 6.07 μA), b (0.950 V, 9.38 μA); (3) with 16 mg·L^−1^ of cysteine at a (0.696 V, 6.34 μA), b (0.962 V, 9.68 μA); (4) with 32 mg·L^−1^ of cysteine at a (0.702 V, 6.68 μA), b (0.985 V, 9.96 μA); (5) with 64 mg·L^−1^ of cysteine at a (0.752 V, 7.25 μA), b (1.01 V, 10.4 μA). Scan rate, 10 mV/s; pulse height 50 mV; pulse time 1 s.

Between 3.69 and 5.33, cysteine shows pH-dependent waves at Au/CFE electrode. The peak current and potential increase with increasing pH, and has a maximum about pH 5.33. On the Au/CFE electrode, the peak potential at 0.686 V, 0.776 V, 1.11 V, 1.12 V, 1.12 V and 1.01 V for pH 3.69, 4.41, 5.33, 6.13, 7.07, and 8.05. It is thought that this was due to an isoelectric point of cysteine (5.02). The peak current of cysteine in phosphate buffer (pH 2.3 and 6.8) is lower than at pH values between 3 and 5. For analytical purposes Briton-Robinson buffer was chosen as the best supporting electrolyte because of its continuous buffering range between pH 4.65 and 5.33. Two anodic waves (at 0.68 V and 0.90 V) were observed in Figure 4. These waves were recorded in less positive potentials than the 0.74 and 1.0 V reported in our previous paper dealing with s ceramic carbon electrode [32]. Therefore, the Au/CFE electrode was chosen for use in the determination of cysteine.

**Figure 5.**
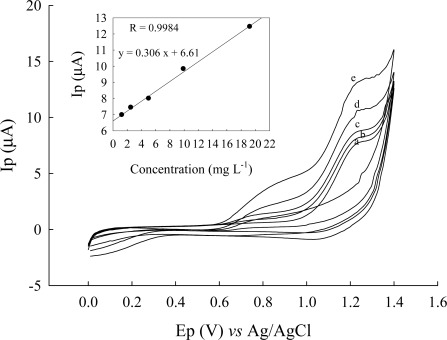
Cyclic voltammograms of cysteine after different concentrations at an Au/CFE electrode and after related current-concentration curve: (**a**) 1.25 mg·L^−1^; (**b**) 2.5 mg·L^−1^; (**c**) 5.0 mg·L^−1^; (**d**) 10 mg·L^−1^; (**e**) 20 mg·L^−1^ in Britton-Robinson buffer (pH 5.33) solution, scan rate at 50 mV/s.

**Figure 6.**
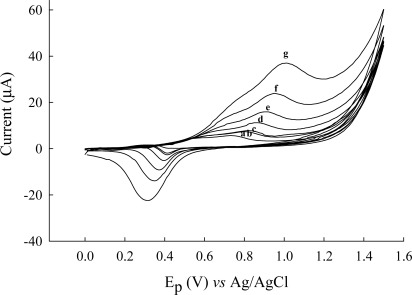
Cyclic voltammograms of cysteine 30.0 mg·L^−1^ in Britton-Robinson buffer (pH 5.33) at various potential scan rates: (**a**) 5 mV/s; (**b**) 10 mV/s; (**c**) 12.5 mV/s; (**d**) 25 mV/s; (**e**) 50 mV/s; (**f**) 100 mV/s (**g**) 200 mV/s.

**Figure 7.**
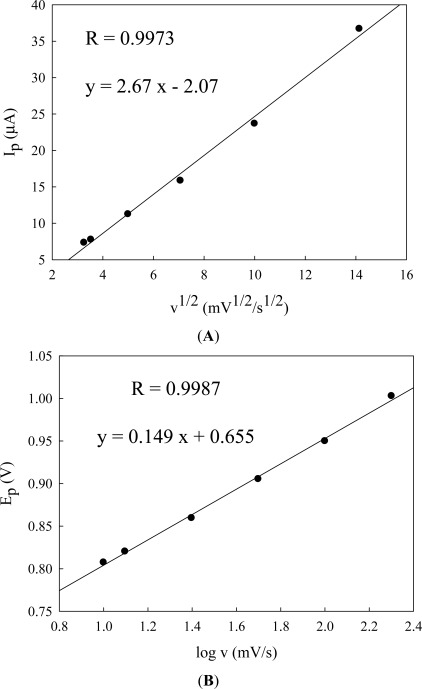
(**A**) Magnitude of the peak current, I_p_, for cysteine oxidation as a function of square root of scan rate and (**B**) peak potentials E_p_ of cysteine oxidation as a function of logarithm of scan rates from Figure 6.

Current-potential curves were plotted using different concentration of cysteine. Experiments were performed at pH 2.81 and 5.33 (results not shown) and pH 3.56 (Figure 5). Cyclic voltammograms of cysteine in Britton-Robinson buffer (pH 3.56) solution at an Au/CFE electrode show one well-defined oxidation (compared to Figure 2 scan rate 10 mV/s) that is due to rapid scan rate 50 mV/s of a portion of the cysteine which diffuses to the electrode surface, and proceeds rapidly as a result of a catalytic effect of the gold. Cyclic voltammograms of different concentrations of cysteine at an Au/CFE electrode are shown in Figure 5, the regression equation being y = 0.306 x + 6.61, the correlation coefficient *r* = 0.9921. The influence of the potential scan rate on the electrochemical response was studied at pH 5.33 (Figure 6). Good linearity was observed between the peak height (current) and the square root of scan rate (v^1/2^) (Figure 7(A)).

The anodic peak current Ip is found to increase with v^1/2^. The relationship between peak potential (Ep) and logarithm of scan rate (log v) (Figure 7(B)) can be used to estimate roughly the number of electrons involved in the catalytic oxidation. From the slope value and by calculating from equation 2.303 RT/αn_a_ F (α the transfer coeffient, and n_a_ the number of electrodes in the rate-determining step), n_a_ = 0.8 (approximately) for an irreversible process. The two-step waves found at pH values between 3 and 8, twice the height of the total wave corresponding to two-electrode oxidation to cystine [31].

### 3.2. Optimum Conditions for Liquid Chromatography-Voltammetric Sensor

Various ratios of methanol-water containing 1.0 mM acetate buffer (pH 4.65) were prepared. After various studies of the retention behavior of the cysteine, baseline separation was achieved. Methanol: water (20:80 v/v) containing 1.0 mM acetate buffer (pH 4.65) was found to be the best eluent for a good sensitivity and higher than the other eluents. Stationary phase was ThermoQuest Hypersil SCX (particle size 5 μm, 250 mm × 4.6 mm i.d.). The detection conditions of the voltammetric detector was operated under pulsed conditions, t_1_ = 180 ms, t_2_ = 180 ms. Initial potential E_1(det)_ = +1.0 V, final potential E_2(ox)_= +2.0 V, flow rate, 0.5 mL/min. Using the injection valve, 20 μL of the prepared standard solution were chromatographed under the operating conditions described above.

**Figure 8.**
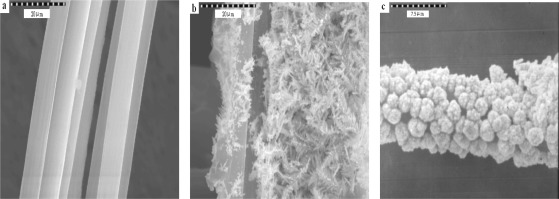
Scanning electron micrographs (at 2 kV) of a Au-coated carbon fibre composite surface. (**a**) un-coated; (**b**) Au deposits (1 mM) 480 s; in 0.1 M perchloric acid (**c**) Au deposits (1 mM) 480 s; in 0.1 M acetate buffer (pH 5.02).

The nature of the deposition conditions primarily affects the specific surface area of the gold catalyst. The optimum conditions for electrochemical deposition of gold have been investigated. The effects of the gold layer were performed by coating the CFE in deposition solution with different times (240–540 s). Electrochemical deposition of Au film on a CFE was achieved in 0.1 M perchloric acid and 0.1 M acetate aqueous solution of 4.0 mM of HAuCl_4_ by repeated potential scan between −1.0 V and +1.0 V (*vs.* Ag/AgCl) (at 100 mV/s), respectively. For comparision of the modified electrode substances, three scanning electron microscope pictures (SEM, JEOL Co.JXA-840) are shown in Figure 8. The Figure 8(a) presents an un-coated carbon fiber i.d. 7.54 μm. As shown in Figure 8(c), gold spherical particles were distributed more uniformly in acetate buffer than the percholic acid (Figure 8(b)).

**Figure 9.**
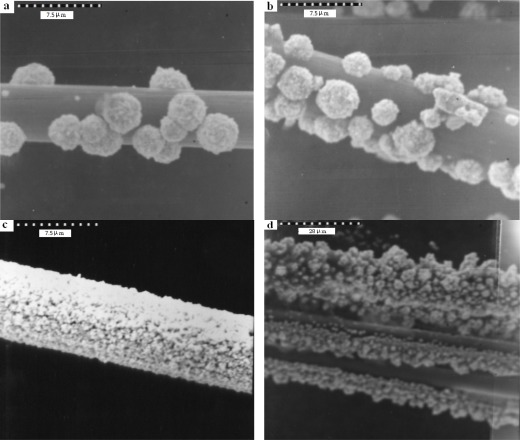
Scanning electron micrographs (at 4 kV) of a Au-coated carbon fiber composite surface. (**a**) Au (4 mM) deposits 240 s; (**b**) Au (4 mM) deposits 360 s; (**c**) Au (4 mM) deposits 480 s; (**d**) Au (4 mM) deposits 540 s in 0.1 M acetate buffer (pH 5.02).

The gold needle-like leaf particles were dispersed with very slight aggregation, as seen in Figure 9(b). A comparision of deposition time and the results are shown in SEM Figure 9(a–d). In Figure 9(c) gold spherical particles were seen and coverage was more uniformly distributed than in the other samples. The particle sizes (Figure 9(a–d)) had diameters of 3.9 μm, 2.5 μm, 0.71 μm and 2.7 μm, respectively. The concentration 4.0 mM of HAuCl_4_ and 480 s of deposition time were used for coating, because the peak height of cysteine was higher than in the other examples.

**Figure 10.**
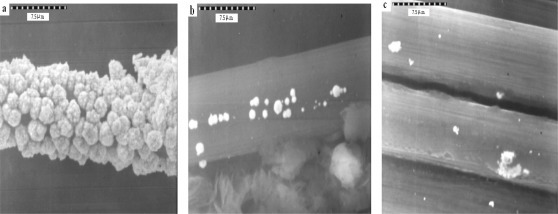
Gold particles distribution in the carbon fiber: (**a**) a bundle of carbon fiber is composed of 8 single fiber; (**b**) a bundle of carbon fiber is composed of 16 single fiber; (**c**) a bundle of carbon fiber is composed of 32 single fiber.

**Figure 11.**
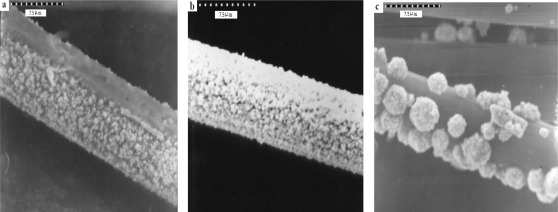
Gold particles distribution on a bundle of carbon fiber is composed of 8 single fiber and their lengths: (**a**) 6 cm; (**b**) 8 cm; (**c**) 12 cm.

The Au particle distribution on the surface of carbon fiber can be affected by the number (Figure 10) and length (Figure 11) of the carbon fibers.

Figure 10 shows that the Au particle distribution on eight single fibers (Figure 10(a)) was more abundant and homogeneous than the others (16 and 32 single fibers; Figure 10(b,c)). Figure 11 shows eight single fibers but of different length (6, 8 and 12 cm). Figure 11(c) shows slight and not homogenous distribution because of the ration of mass diffusion to long length.

In Table 1, the retention time and peak height as functions of the fiber length are given. The retention time is independent of detector length. It can be seen that the 8 cm detector is the most suitable since the peak height of cysteine is the highest than the others. Therefore, the CFE (length 8 cm) was chosen to deposit Au for use in the determination of cysteine.

The retention time and peak height are dependent on the mobile phase flow-rate and varies from 0.2–0.6 mL·min^−1^ (Table 2).

**Figure 12.**
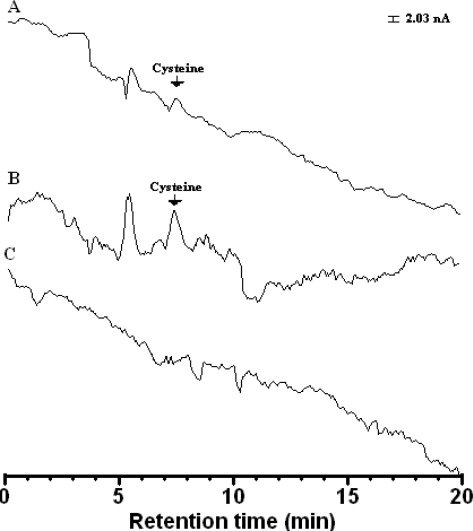
Chromatograms obtained using Au electrode (**A**) and Au/CFE (**B**) for cysteine (0.5 mg·L^−1^) and (**C**) blank solution. Conditions: electrode, Au–modified carbon fiber detector (length: 8 cm); stationary phase, ThermoQuest Hypersil SCX (particle size 5 μm, 250 mm × 4.6 mm i.d.); Mobile phase, methanol: water (20:80 v/v) containing 1.0 mM acetate buffer (pH 4.65); detection conditions: pulsed conditions, t_1_ =180 ms, t_2_ = 180 ms. Initial potential E_1(det)_ = +1.0 V, final potential E_2(ox)_= +2.0 V, flow rate, 0.5 mL·min^−1^.

It is apparent that the flow rate 0.5 mL·min^−1^ is most suitable, because the peak height of cysteine is the highest and retention time of 7.15 min is shorter than the others (except 0.6 mL·min^−1^). The chromatograms in Figure 12(A–C) are comparable to a chromatogram of cysteine at bare Au, Au/CFE and blank solution. The peak height of cysteine at Au electrode (retention time 7.49 min) is smaller than that on Au/CFE (retention time 7.40 min). The Au electrode is expensive and needs a clean surface which cannot be discarded as Au/CFE. Therefore, the Au/CFE was suitable as working electrode in a flow cell-voltammetric sensor for the determination of cysteine.

### 3.3. Stability of Flow Cell-Voltammetric Sensor

**Figure 13.**
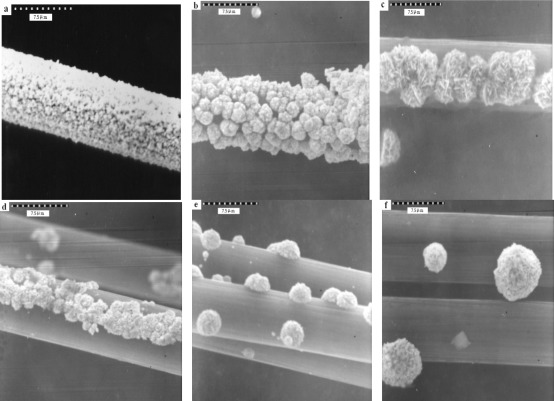
Gold particle distribution in the Au -coated carbon fiber as the working electrode in the flow cell after (**a**) 0 h; (**b**) 2 h; (**c**) 5 h; (**d**) 7 h; (**e**) 9 h; (**f**) 12 h.

The operational stability of the sensors was studied by continuous exposure to the flow stream. Figure 13 shows the stability of the sensor over 12 h of repetitive injections. The sensor was run with an interval time of 30 min for every injection. The Au/CFE can be used average 12–15 times and after 9 h ceases to perform better than a bare CFE. The presence of a few gold spherical particles was observed on the SEM images over 9 h (Figure 13(e)). This is due the flow assumptions, the dispersion and hydrodynamic effects would predict SEM pattern, *i.e.*, Au particles decrease as the flow time in the flow cell increases at a flow rate 0.5 mL·min^−1^.

**Figure 14.**
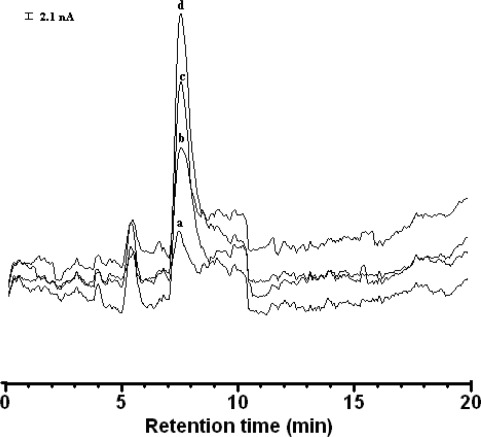
The LC-ECD chromatograms recorded to produce analytical curves for cysteine. Peaks; (**a**) 1.0 mg·L^−1;^ (**b**) 2.0 mg·L^−1;^ (**c**) 3.0 mg·L^−1;^ (**d**) 4.0 mg·L^−1^. Liquid chromatography-electrochemical detection analysis conditions were identical to those listed in Figure 12.

The proposed LC-PAD method was applied to the determination of cysteine and the resulting chromatograms are shown in Figure 14. The calibration curve showed good linearity over the range 0.5–4.0 mg·L^−1^; the regression equation was *y* =169 *x* + 24.4, and the correlation coefficient was *r* = 0.9984. The limits of quantification for cysteine was below 60 ng·m·L^−1^.

## 4. Conclusions

In this article, we report the construction of gold-containing deposited modified carbon fiber electrodes, and their application as voltammetric sensors in the liquid chromatography-pulsed amperometric detection (LC-PAD) determination of cysteine. The film of Au/CFE electrode was characterized by cyclic voltammetry and SEM. Electrodes formed via this modified approach not only exhibited more activity toward this analyte, but also provided stable, quantitatively reproducible performance in the chromatographic stream. Thus, the proposed analytical method offers a valid alternative to absorbance or fluorescence spectrometry detection of cysteine where derivatization procedures are needed.

## Figures and Tables

**Table 1.** Dependences of retention time and peak height cysteine (2.5 mg·L^−1^) on the carbon fiber detector working length.

**CFE length (cm)**	**Retention time (min)**	**Peak height (mV)**
6	7.17	60.3
8	7.15	224
10	7.08	197
12	7.07	135

**Table 2.** Dependences of retention time and peak height of cysteine (2.5 mg·L^−1^) on the flow rate (mL·min^−1^) at carbon fiber detector (length 8 cm).

**Flow rate (mL·min^−1^)**	**Retention time (min)**	**Peak height (mV)**
0.2	17.0	445
0.3	11.4	449
0.4	8.54	425
0.5	7.15	507
0.6	5.70	327
